# The use of Kampo medications that may cause heart failure in hospitalized acute heart failure patients in a Japanese hospital

**DOI:** 10.1002/jgf2.411

**Published:** 2020-12-09

**Authors:** Junpei Komagamine, Miho Kaminaga, Toshikazu Omori, Shinpei Tatsumi

**Affiliations:** ^1^ Department of Internal Medicine National Hospital Organization Tochigi Medical Center Utsunomiya Japan; ^2^ Department of Pharmacy National Hospital Organization Tochigi Medical Center Utsunomiya Japan

**Keywords:** heart failure, herbal medicine, Kampo medications, licorice

## Abstract

**Background:**

The use of Kampo medications (Japanese traditional herbal medications) is common in Japan. However, some Kampo medications may cause heart failure. Given that the incidence of heart failure has increased in past decades, investigating the prevalence of the use of Kampo medications that may cause heart failure in patients with acute heart failure is important.

**Method:**

A retrospective cross‐sectional study was conducted. All 437 consecutive hospitalized patients with acute heart failure from April 2017 to October 2019 were included. The primary outcome was the use of Kampo medications, including ephedra, licorice, aconite, or ginseng, which were defined as those that may cause heart failure. The causality between these medications and the index of acute heart failure was determined by clinical pharmacists based on the Naranjo criteria.

**Results:**

The mean patient age was 81.1 years old, and 199 (54.5%) were women. Kampo medications that may cause heart failure were used in 30 patients (6.9%), and in four of these patients, acute heart failure was judged to be caused by Kampo medications. In the multivariable analysis, the number of non‐Kampo medications used regularly (OR 1.13) and female sex (OR 2.23) were the only independent predictive factors for the use of Kampo medications that may cause heart failure.

**Conclusions:**

A substantial proportion of acute heart failure patients in Japanese hospitals use Kampo medications that may cause heart failure. Further study is warranted to investigate the causal link between the incidence of acute heart failure and the use of these herbal medications.

## Graphical abstract

Kampo medications composed of ephedra, licorice, aconite, or ginseng may cause or exacerbate heart failure. Our findings suggest that acute heart failure may be caused by these herbal medications in a substantial proportion of patients hospitalized with acute heart failure in a Japanese hospital.

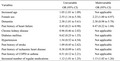

## 1 INTRODUCTION

Kampo medicine (Japanese traditional herbal medicine) has developed into a unique form of medicine in Japanese practice and culture since it was introduced in Japan 1500 years ago.^1^, ^2^, ^3^ It has been integrated into the Japanese national healthcare system; 148 Kampo extract formulations and 187 types of crude drugs are approved and used under the national health insurance program.^4^ In Kampo medicine, extract formulations are preferentially used as fundamental drugs over single crude extracts.^5^ Kampo medications are available over‐the‐counter (OTC) and are the most frequently used complementary and alternative medicine therapies prescribed by physicians in Japan.^6^ Based on an Internet‐based survey of physicians,^7^ the most common diseases for which physicians prescribe Kampo medications are muscle cramps, followed by acute respiratory tract infections, constipation, and nonspecific complaints and menopausal symptoms. Nonetheless, the inappropriate use of prescribed Kampo medications might be common in Japan.^8^

Certain components of Kampo medications can be harmful to cardiac function.^9^, ^10^, ^11^, ^12^, ^13^, ^14^, ^15^, ^16^ For example, licorice can cause heart failure through sodium retention.^10^, ^11^ Therefore, the use of licorice should be suspected as a cause in patients with newly diagnosed acute heart failure.^11^ Moreover, ephedra is also associated with adverse cardiovascular events.^12^, ^13^ Therefore, recent guidelines have reported that certain herbal medications, such as ephedra and licorice, can cause or exacerbate heart failure.^17^ Four of the 5 Kampo medications with the highest production value in Japan include licorice.^18^ Heart failure is common, particularly in the elderly population.^19^ Although few side effects of heart failure due to Kampo medications have been reported in the Japanese adverse drug event report database,^20^, ^21^ adverse drug reactions are often unrecognized by physicians,^22^ and more than 90% of adverse drug events are not reported.^23^ Therefore, monitoring the use of these herbal medications in patients in Japan with acute heart failure is important.

Past studies conducted in countries outside Japan have reported that 2% to 46% of patients with chronic heart failure or cardiac disease used herbal medications.^24^ However, these past studies^25^, ^26^, ^27^, ^28^, ^29^, ^30^ did not investigate the causal link between herbal medication use and the incidence of heart failure. Moreover, no studies have been conducted to determine the prevalence of the use of herbal medications that can cause or exacerbate heart failure in patients with acute heart failure.

Thus, the aim of this study was to investigate the prevalence of the use of Kampo medications that can cause or exacerbate heart failure (hereafter denoted CEHF Kampo medications) in hospitalized patients with acute heart failure in Japan. The causal link between the use of CEHF Kampo medications and acute heart failure was also investigated.

## 2 METHODS

### 2.1 Study setting and design

A retrospective, single‐center, cross‐sectional study using electronic medical records was conducted to investigate the prevalence of the use of CEHF Kampo medications among hospitalized patients with acute heart failure. Our hospital is an acute care hospital. This research was approved by the institutional medical ethical committee.

### 2.2 Inclusion and exclusion criteria

All consecutive patients aged 18 years old or older who were hospitalized due to acute heart failure from April 2017 to October 2019 were included. Patients with missing information about medications before the index admission were excluded. Acute heart failure was defined as either new‐onset heart failure or decompensation of chronic established heart failure with symptoms sufficient to lead to hospitalization based on recent guidelines.^31^ During the study period, a total of 5157 patients were hospitalized in the internal medicine ward of our hospital. Of those, 439 patients met the inclusion criteria. After excluding two patients without information on medications before the index admission, 437 patients were included in the final analysis.

### 2.3 Screening and data collection

Information on patient age, sex, Charlson Comorbidity Index score.^32^ past medical history, vital signs, laboratory data, triggers of the index acute heart failure, and prognosis was collected by reviewing all available electronic medical records from September 2014 to the time of the index admission. However, for chronic kidney disease, patients whose estimated creatinine clearance was less than 60 ml/min/1.73 m^2^ for 3 months were also judged to have chronic kidney disease even if chronic kidney disease was not documented in the medical records. Information about prescribed drugs, including Kampo medications, was obtained from a comprehensive medication list compiled by pharmacists during the course of routine care. Information about triggers of acute heart failure was collected from medical records documented by the physicians caring for the patients.

### 2.4 Outcome measures

The primary outcome was the proportion of all acute heart failure patients who used CEHF Kampo medications. Based on a recent scientific statement,^17^ any Kampo medications that include ephedra,^12^, ^13^ aconite,^14^, ^15^ ginseng,^23^ or licorice^10^, ^11^ were defined as CEHF Kampo medications (Table 1 and Table S1). We included only prescribed Kampo medications because information about OTC medications collected in routine care is inaccurate.

**Table 1:** Kampo medications containing ephedra, aconite, ginseng, or licorice used for the 437 hospitalized patients with acute heart failure

Ingredient	Kampo medications
Ephedra	*Shoseiryto, Maoto*
Aconite	*Goshajinkigan*
Ginseng	*Juzentaihoto, Daikenchuto, Keishininjinto, Rikkunshito, Bakumondoto, Mokubioto, Byakkokaninjinto, Goshuyuto, Ninjinyoeito, Hochuekkito*
Licorice	*Shakuyakukanzoto, Junchoto, Yokukansan, Juzentaihoto, Keishininjinto, Rikkunshito, Yokukansankachinpihange, Shoseiryuto, Bakumondoto, Byakkokaninjinto, Ryokeijutukanto, Ninjinyoeito, Hochuekkito, Maoto*

In acute heart failure patients who used CEHF Kampo medications, causality was assessed according to the Naranjo criteria (Table S2). Three pharmacists independently assessed these cases through chart reviews and determined the total score. The causality was assigned to a probability category from the total score as follows: “definite,” 9 or greater; “probable,” 5 to 8; “possible,” 1 to 4; and “doubtful,” less than 1. The index acute heart failure was judged to be caused by CEHF Kampo medications if there was a “definite” or “probable” causal association based on the Naranjo criteria. Disagreements among the reviewers were resolved by discussion. Detailed results of this assessment are shown in a Table S3.

### 2.5 Statistical analysis

There were no past studies reporting the prevalence of CEHF Kampo medications among patients hospitalized due to acute heart failure. Therefore, assuming that the proportion of patients who used CEHF Kampo medications was 6%, based on a previous study to determine the prevalence of potentially inappropriate use of Kampo medications among elderly patients in outpatient settings,^8^ approximately 400 acute heart failure patients were needed to provide a precision of 3% for the calculation of the 95% confidence intervals (CIs) of the primary outcomes.

Descriptive statistics were used to report the baseline characteristics of the study population. To identify the independent risk factors associated with the use of CEHF Kampo medications, multivariable analysis with binary logistic regression was conducted. We examined the associations between the primary outcome and age, sex, past medical history, and number of regular non‐Kampo medications. Then, the variables that had p‐values of 0.2 or more were removed using the backward method. The level of statistical significance was set at 5%. These analyses were performed with STATA software, version 15 (LightStone, Tokyo, Japan).

## 3 RESULTS

The baseline characteristics of the 437 patients who were hospitalized due to acute heart failure are shown in Table 2 (detailed information is shown in the Table S4). The mean patient age was 81.1 years old (range, 30‐103), and 199 (45.5%) patients were women. Moreover, the mean Charlson Comorbidity Index score was 2.4 (Standard Deviation (SD) = 1.7), 62 (14.2%) patients had dementia, and 239 (54.7%) had a past history of heart failure. The mean number of regular non‐Kampo medications was 6.2 drugs (SD = 3.4). With regard to vital signs at admission, the mean systolic blood pressure was 146 mm Hg, the mean diastolic blood pressure was 86 mm Hg, and the mean heart rate was 96 beats per minute. With regard to the laboratory findings, the mean serum sodium concentration was 140 mEq/L, the mean serum potassium concentration was 4.3 mEq/L, the mean serum creatinine level was 1.3 mg/dL, and the mean serum brain natriuretic peptide level was 1232 ng/mL. The most commonly documented triggers for the index acute heart failure were diet (n = 100, 22.9%), followed by arrhythmia (n = 75, 17.2%) and infection (n = 36, 8.2%). However, no triggers were identified in 120 (27.5%) of the patients.

**Table 2:** Baseline characteristics of the 437 hospitalized patients with acute heart failure^a^

Characteristics	Total (n = 437)	Use of Kampo medications that may cause or exacerbate heart failure	
		No (n = 407)	Yes (n = 30)
Mean patient age, SD	81.1 (12.1)	80.7 (12.3)	86.0 (7.8)
Female sex	199 (45.5)	179 (44.0)	20 (66.7)
Japanese nationality	435 (99.5)	405 (99.5)	30 (100.0)
Nursing home resident	59 (13.5)	54 (13.3)	5 (16.7)
Mean charlson comorbidity index score, SD	2.4 (1.7)	2.4 (1.7)	2.0 (1.6)
Past medical history			
Stroke	96 (22.0)	89 (21.9)	7 (23.3)
Dementia	62 (14.2)	54 (13.3)	8 (26.7)
Diabetes mellitus	123 (28.2)	117 (28.8)	6 (20.0)
Ischemic heart disease	66 (15.1)	64 (15.7)	2 (6.7)
COPD or asthma	52 (11.9)	50 (12.3)	2 (6.7)
Heart failure	239 (54.7)	228 (56.0)	11 (36.7)
Hypertension	355 (81.2)	329 (80.8)	26 (86.7)
Chronic kidney disease	208 (47.6)	194 (47.7)	14 (46.7)
Mean number of regular medications, SD	6.2 (3.4)	6.1 (3.4)	7.4 (3.5)
Medication use			
NSAIDs	23 (5.3)	22 (5.4)	1 (3.3)
Beta‐blockers	141 (32.3)	137 (33.7)	4 (13.3)
ACE inhibitor or ARB	200 (45.8)	185 (45.5)	15 (50.0)
Loop diuretics	226 (51.7)	210 (51.6)	16 (53.3)
Spironolactone	64 (14.7)	60 (14.7)	4 (13.3)
Digoxin	12 (2.8)	11 (2.7)	1 (3.3)
Laboratory findings at admission			
Hemoglobin, g/dL	11.8 (2.1)	11.8 (2.2)	11.7 (1.8)
Blood urea nitrogen, mg/dL	29.2 (16.8)	29.2 (16.9)	30.0 (14.4)
Creatinine, mg/dL	1.3 (0.7)	1.3 (0.7)	1.2 (0.8)
Sodium, mmol/L	140 (8)	140 (8)	141 (5)
Potassium, mmol/L	4.3 (0.7)	4.3 (0.7)	4.1 (0.6)
Brain natriuretic peptide^b^, ng/mL	1233 (1100)	1246 (1119)	1054 (813)
Left ventricular ejection fraction^c^, SD	45 (17)	45 (17)	46 (19)
Triggers of heart failure^d^			
Diet	100 (22.9)	93 (22.9)	7 (23.3)
Arrhythmia	75 (17.2)	69 (17.0)	6 (20.0)
Uncontrolled hypertension	46 (10.5)	45 (11.1)	1 (3.3)
Infection	36 (8.2)	32 (7.9)	4 (13.3)
Cardiac ischemia	28 (6.4)	28 (6.9)	0 (0.0)
Drug induced	19 (4.4)	18 (4.4)	1 (3.3)
Drug adherence	16 (3.7)	15 (3.7)	1 (3.3)
Anemia	10 (2.3)	8 (2.0)	2 (6.7)
Exacerbation of CKD	6 (1.4)	6 (1.5)	0 (0.0)
Unknown	120 (27.5)	112 (27.5)	8 (26.7)
In‐hospital death	44 (10.1)	40 (9.8)	4 (13.3)

Abbreviations: ACE, angiotensin‐converting enzyme; ARB, angiotensin receptor blocker; CKD, chronic kidney disease; NSAIDs, nonsteroidal anti‐inflammatory drugs; SD, standard deviation.

^a^ Values are expressed as the numbers with the percentages of the total numbers unless otherwise stated.

^b^ Excludes 10 patients who were not tested for brain natriuretic peptide.

^c^ Excludes 5 patients who were not evaluated for left ventricular ejection fraction.

^d^ Based on judgment by physicians caring for the patient.

Kampo medications that can cause or exacerbate heart failure were used in 30 patients (6.9%; 95% CI, 4.5%‐9.3%). Among these patients, the most common prescribed herbal medication was licorice (n = 28, 93.3%), followed by ginseng (n = 12, 40.0%) and ephedra (n = 3, 10.0%) (Table 3). Based on the causality assessment between CEHF Kampo medications and the index acute heart failure based on the Naranjo criteria, four cases of heart failure were judged to be caused by CEHF Kampo medications.

**Table 3:** Characteristics of Kampo medications that may cause heart failure^a^ and were used in 30 acute heart failure patients^b^

	Total (n = 30)
Use of Kampo medications that may cause heart failure^a^, ^c^	
Regular	29 (96.7)
As‐needed	2 (6.7)
Kampo medications^c^	
Licorice composition	28 (93.3)
Ginseng composition	12 (40.0)
Ephedra composition	3 (10.0)
Aconite composition	1 (3.3)

^a^ These indicate any Kampo medications composed of licorice, ginseng, ephedra, or aconite.

^b^ Values are expressed as the number and percentage of the total number.

^c^ One patient could be given more than one medication.

In the univariable analysis, increased age, female sex, dementia, and use of a large number of regular non‐Kampo medications were significantly associated with increased risk for the use of CEHF Kampo medications, while a past history of heart failure was significantly associated with a decreased risk (Table 4). However, multivariable analysis revealed that female sex (odds ratio (OR) 2.23; 95% CI, 1.00 to 4.97) and the use of a large number of regular non‐Kampo medications (OR 1.13; 95% CI, 1.02‐1.26) were the only independent predictive factors associated with the use of CEHF Kampo medications.

**Table 4:** Results of univariable and multivariable analyses of the predictive factors associated with the use of Kampo medications that may cause heart failure

Variables	Univariable^a^ OR (95% CI)	Multivariable^a^, ^b^ OR (95% CI)
Increased age^c^, per year	1.05 (1.01‐1.09)*	Not applicable
Female sex	2.55 (1.16‐5.58)*	2.23 (1.00‐4.97)*
Dementia	2.38 (1.01‐5.61)*	2.38 (0.98‐5.78)
Past history of heart failure	0.45 (0.21‐0.98)*	0.47 (0.22‐1.04)
Chronic kidney disease	0.96 (0.46‐2.02)	Not applicable
Diabetes mellitus	0.62 (0.25‐1.55)	Not applicable
Hypertension	1.54 (0.52‐4.54)	Not applicable
Past history of stroke	1.09 (0.45‐2.62)	Not applicable
Past history of ischemic heart disease	0.38 (0.09‐1.65)	Not applicable
Past history of COPD or asthma	0.51 (0.12‐2.21)	Not applicable
Increased number of regular medications^c^, ^d^, per one medication	1.12 (1.01‐1.25)*	1.13 (1.02‐1.26)*

Abbreviations: CI, confidence interval; COPD, chronic obstructive pulmonary disease; OR, odds ratio.

^a^ The analyses were conducted for the 437 hospitalized patients with acute heart failure. The level of statistical significance was set at *P* < .05. Asterisks indicate a significant association between the selected variables and the use of Kampo medications that may cause heart failure.

^b^ Age, sex, medical history, and number of regular non‐Kampo medications were included as variables. Then, the variables that had a *p*‐value of 0.2 or more were removed via the backward method.

^c^ Continuous variable was used.

^d^ Kampo medications were not included.

## 4 DISCUSSION

This study was the first to determine the prevalence of the use of herbal medications that can cause or exacerbate heart failure in patients hospitalized with acute heart failure. The prevalence of the use of Kampo medications containing ginseng in the present study was 2.7%, which was similar to the prevalence of the use of ginseng among patients with chronic heart failure in past studies conducted outside of Japan.^24^, ^25^, ^26^, ^27^, ^28^ However, there was no use of licorice, ephedra, or aconite reported in previous studies^24^, ^25^, ^26^, ^27^, ^28^, ^29^ investigating the use of herbal remedies in patients with chronic heart failure. Therefore, the prevalence of the use of these herbal medications that can cause or exacerbate heart failure in this study was unexpectedly high, although the target patients in the present study were different from those in previous studies.^24^, ^25^, ^26^, ^27^, ^28^, ^29^ Moreover, it is problematic that approximately one‐third of acute heart failure patients who took any CEHF Kampo medications already had a history of heart failure. This finding means that these Kampo medications are being prescribed to some patients with chronic heart failure who should avoid using them if possible. In addition, the multivariable analysis revealed that the use of CEHF Kampo medications is more common in patients taking a large number of regular non‐Kampo medications. The use of Kampo medications in patients currently using a large number of medications is more likely to cause herb‐drug interactions, which can result in harmful events.^33^, ^34^ Therefore, the use of herbal medications should be minimized in patients with polypharmacy. Thus, our findings suggest that some efforts to minimize the use of Kampo medications that can be harmful to cardiac function among heart failure patients with polypharmacy are needed in Japan. A strategy for improving potentially inappropriate use of Kampo medications would likely be similar to a strategy for improving inappropriate use of non‐Kampo medications.^35^ Therefore, interventions such as pharmacist‐led medication reviews would be useful.

Female sex and the use of a large number of regular non‐Kampo medications were the only independent risk factors associated with the use of CEHF Kampo medications in the present study. This result was similar to that of studies of Western medications reporting that female sex and number of regular medications were significant factors associated with potentially inappropriate medication for elderly patients.^36^ The use of a larger number of medications reflects the presence of more severe comorbidities. Therefore, the higher medical needs for Kampo medications might result in the use of CEHF Kampo medications. A past Japanese study reported that women used more complementary alternative medicine, including Kampo medications, than men.^37^ Therefore, the frequent use of Kampo medications by women might be one of the reasons why women were more likely than men to use the CEHF Kampo medications.

In the current study, the most common CEHF Kampo medications were Kampo formulations containing licorice. Given the sodium retention caused by licorice,^10^, ^11^ the use of licorice should be avoided if possible. As mentioned previously, however, most of the Kampo medications used in Japan include licorice.^18^ Our findings indicate that Kampo medications, particularly those containing licorice, should be minimized for patients at high risk for heart failure. Furthermore, the use of licorice should be suspected as one of the causes for patients with newly diagnosed acute heart failure.^11^

Based on the Naranjo criteria, most of the CEHF Kampo medications used in this study were relatively less likely to cause acute heart failure. However, this result should be interpreted cautiously. Triggers of acute heart failure are often multifactorial.^38^ Indeed, the presence of other factors associated with adverse events decreases the association between the drug and adverse events according to the Naranjo criteria. For example, a score based on the Naranjo criteria can be reduced by one point for the concomitant use of nonsteroidal anti‐inflammatory drugs. Therefore, the presence of other triggers for heart failure might underestimate the strength of the causal link between the use of Kampo medications and the index acute heart failure.

The strength of our study is that we included all consecutive hospitalized patients with acute heart failure. Therefore, our findings represent a real‐world situation. In addition, more than two pharmacists independently assessed the causal link between the use of CEHF Kampo medications and heart failure according to the standard criteria. However, our findings should be interpreted with caution. First, our study was a retrospective, observational study. Therefore, the data extracted for use in this study might not be accurate. Second, given that the attitude toward prescribing Kampo medications is different between Japanese physicians,^7^ a regional or institutional difference might largely affect our outcomes. Therefore, the single‐center design limited the generalizability of our results. Third, we excluded OTC Kampo medications. Thus, the prevalence of the use of CEHF Kampo medications might have been underestimated. Fourth, we investigated only Kampo medications containing ephedra, aconite, ginseng, or licorice, which may have underestimated the prevalence of heart failure caused by Kampo medications. Fifth, the number of occurrences of the primary outcome was small in the present study. This limits the accuracy of our estimate from the multivariable analysis. Sixth, we assessed causality according to the Naranjo criteria. However, there is no gold standard for the assessment of the causal link between the use of CEHF Kampo medications and the occurrence of adverse events.^39^ Moreover, there have been limited data about the adverse effects of aconite and ginseng on the cardiovascular system, although recent guidelines report that these ingredients may be harmful in patients with heart failure.^17^ In addition, a recent randomized controlled trial reported beneficial effects of *mokuboito*, which is a Kampo medication containing ginseng and sinomenine, on heart failure symptoms, although its beneficial effect is believed to be attributed to sinomenine, not ginseng.^40^ Therefore, it is possible that the prevalence of acute heart failure due to the use of CEHF Kampo medications might have been overestimated.

In conclusion, one in every 14 patients hospitalized in a Japanese hospital with acute heart failure used CEHF Kampo medications. Most were Kampo medications that contained licorice. Given the limited generalizability due to the single‐center design, our findings must be confirmed in other Japanese hospitals. Moreover, further studies are warranted to investigate the causal link between the use of these herbal medications and the incidence of acute heart failure.

## CONFLICT OF INTEREST

The authors have stated explicitly that there are no conflicts of interest in connection with this article.

## Supporting information

App S1

## References

[1] Motoo Y , Seki T , Tsutani K . Traditional Japanese medicine, Kampo: its history and current status. Chin J Integr Med. 2011;17(2):85–7. 10.1007/s11655-011-0653-y 21390572

[2] Watanabe K , Matsuura K , Gao P , Hottenbacher L , Tokunaga H , Nishimura K , et al. Traditional Japanese Kampo medicine: clinical research between modernity and traditional medicine‐the state of research and methodological suggestions for the future. Evid Based Complement Alternat Med. 2011;2011:513842. 10.1093/ecam/neq067 21687585 PMC3114407

[3] Yu F , Takahashi T , Moriya J , Kawaura K , Yamakawa J , Kusaka K , et al. Traditional Chinese medicine and Kampo: a review from the distant past for the future. J Int Med Res. 2006;34(3):231–9. 10.1177/147323000603400301 16866016

[4] Shimada Y , Fujimoto M , Nogami T , Watari H , Kitahara H , Misawa H , et al. Patient safety incident reports related to traditional Japanese Kampo medicines: medication errors and adverse drug events in a university hospital for a ten‐year period. BMC Complement Altern Med. 2017;17(1):547. 10.1186/s12906-017-2051-2 29268743 PMC5740942

[5] Arai I , Kawahara N . Kampo pharmaceutical products in the Japanese health‐care system: Legal status and quality assurance. Tradit Kampo Med. 2019;6(1):3–11. 10.1002/tkm2.1204

[6] Fujiwara K , Imanishi J , Watanabe S , Ozasa K , Sakurada K . Changes in attitudes of Japanese doctors toward complementary and alternative medicine‐comparison of surveys in 1999 and 2005 in Kyoto. Evid Based Complement Altern Med. 2011;2011:608921. 10.1093/ecam/nep040 PMC3136716 19465404

[7] Japan Kampo Medicine Manufacturers Association . Survey of Kampo medicine prescription 2011 (in Japanese). https://www.nikkankyo.org/serv/pdf/jittaichousa2011.pdf. Accessed 28 February 2020.

[8] Komagamine J , Hagane K . Prevalence of the potentially inappropriate Kampo medications to be used with caution among elderly patients taking any prescribed Kampo medications at a single centre in Japan: a retrospective cross‐sectional study. BMC Complement Altern Med. 2018;18(1):155. 10.1186/s12906-018-2228-3 29751840 PMC5948909

[9] A World Health Organization . WHO Monographs on Selected Medicinal Plants – 1. http://apps.who.int/medicinedocs/en/d/Js2200e/20.html. Accessed 28 February 2020.

[10] Conn JW , Rovner DR , Cohen EL . Licorice‐induced pseudoaldosteronism. Hypertension, hypokaremia, aldosteronopenia, and suppressed plasma renin activity. JAMA. 1968;205(7):492–6. 5695305 10.1001/jama.205.7.492

[11] Chamberlain TJ . Licorice poisoning, pseudoaldosteronism, and heart failure. JAMA. 1970;213(8):1343. 5468336

[12] Haller CA , Benowitz NL . Adverse cardiovascular and central nervous events associated with dietary supplements containing ephedra alkaloids. N Engl J Med. 2000;343:1833–8. 11117974 10.1056/NEJM200012213432502

[13] Peters CM , O'Neill JO , Young JB , Bott‐Silverman C . Is there an association between ephedra and heart failure? a case series. J Card Fail. 2005;11(1):9–11. 15704057 10.1016/j.cardfail.2004.04.003

[14] Tai YT , But PP , Young K , Lau CP . Cardiotoxicity after accidental herb‐induced aconite poisoning. Lancet. 1992;340:1254–6. 1359321 10.1016/0140-6736(92)92951-b

[15] Lin CC , Chan TY , Deng JF . Clinical features and management of herb‐induced aconitine poisoning. Ann Emerg Med. 2004;43:574–9. 15111916 10.1016/j.annemergmed.2003.10.046

[16] Paik DJ , Lee CH . Review of cases of patient risk associated with ginseng abuse and misuse. J Ginseng Res. 2015;39(2):89–93. 26045681 10.1016/j.jgr.2014.11.005 PMC4452531

[17] Pagell RL 2nd , O'Bryant CL , Cheng D , Dow TJ , Ky B , Stein CM , et al. Drugs that may cause of exacerbate heart failure: a scientific statement from the American Heart Association. Circulation. 2016;134(6):e32–69. 10.1161/CIR.0000000000000426 27400984

[18] Japan Kampo Medicine Manufacturers Association . Production dynamics of Kampo preparations 2018 (in Japanese). https://www.nikkankyo.org/serv/movement/h30/all.pdf. Accessed 28 February 2020.

[19] van Riet EE , Hoes AW , Wagenaar KP , Limburg A , Landman MA , Rutten FH . Epidemiology of heart failure: the prevalence of heart failure and ventricular dysfunction in older adults over time. Eur J Heart Fail. 2016;18(3):242–52. 10.1002/ejhf.483 26727047

[20] Shimada Y , Fujimoto M , Nogami T , Watari H . Adverse events associated with ethical Kampo formulations: analysis of the domestic adverse‐event data reports of the Ministry of Health, Labor, and Welfare in Japan. Evid Based Complement Alternat Med. 2019;2019:1643804. 31118950 10.1155/2019/1643804 PMC6500660

[21] Arai I , Harada Y , Koda H , Tsutani K , Motoo Y . Estimated incidence per population of adverse drug reactions to Kampo medicines from the Japanese adverse drug event report database (JADER). Tradit Kampo Med. 2020;7:3–16.

[22] Roulet L , Ballereau F , Hardouin JB , Chiffoleau A , Moret L , Potel G , et al. Assessment of adverse drug event recognition by emergency physicians in a French teaching hospital. Emerg Med J. 2013;30:63–7. 22366041 10.1136/emermed-2011-200482

[23] Hazell L , Shakir SA . Under‐reporting of adverse drug reactions: a systematic review. Drug Saf. 2006;29(5):385–96. 16689555 10.2165/00002018-200629050-00003

[24] Grant SJ , Bin YS , Kiat H , Chang DHT . The use of complementary and alternative medicine by people with caridiovascular disease: a systematic review. BMC Public Health. 2012;12:299. 22536991 10.1186/1471-2458-12-299 PMC3444368

[25] Ackman ML , Campbell JB , Buzak KA , Tsuyuki RT , Montague TJ , Teo KK . Use of nonprescription medication by patients with congestive heart failure. Ann Pharmacother. 1999;33(6):674–9. 10.1345/aph.18283 10410177

[26] Zick SM , Blume A , Aaronson KD . The prevalence and pattern of complementary and alternative supplement use in individuals with chronic heart failure. J Card Fail. 2005;11(8):586–9. 10.1016/j.cardfail.2005.06.427 16230260

[27] Corso ED , Bondiani AL , Zanolla L , Vassanelli C . Nurse educational activity on non‐prescription therapies in patients with chronic heart failure. Eur J Cardiovasc Nurs. 2007;6(4):314–20. 10.1016/j.ejcnurse.2007.04.001 17512802

[28] Albert NM , Rathman L , Ross D , Walker D , Bena J , McIntyre S , et al. Predictors of over‐the‐counter drug and herbal therapies use in elderly patients with heart failure. J Card Fail. 2009;15(7):600–6. 10.1016/j.cardfail.2009.02.001 19700137

[29] Mattila M , Boehm L , Burke S , Kashyap A , Holschbach L , Miller T , et al. Nonprescription medication use in patients with heart failure: assessment methods, utilization, and patterns, and discrepancies with medical records. J Card Fail. 2013;19(12):811–5. 10.1016/j.cardfail.2013.10.009 24184371

[30] Martínez‐Sellés M , Robles JAG , Muñoz R , Serrano JA , Frades E , Muñoa MD , et al. Pharmacological treatment in patients with heart failure: patients knowledge and occurrence of polypharmacy, alternative medicine and immunizations. Eur J Heart Fail. 2004;6(2):219–26. 10.1016/j.ejheart.2003.09.009 14984730

[31] Ponikowski P , Voors AA , Anker SD , Bueno H , Cleland JGF , Coats AJS , et al. 2016 ESC Guidelines for the diagnosis and treatment of acute and chronic heart failure: The Task Force for the diagnosis and treatment of acute and chronic heart failure of the European Society of Cardiology (ESC). Developed with the special contribution of the Heart Failure Association (HFA) of the ESC. Eur Heart J. 2016;37(27):2129–200. 10.1093/eurheartj/ehw128 27206819

[32] Charlson ME , Pompei P , Ales KL , MacKenzie CR . A new method of classifying prognostic comorbidity in longitudinal studies: development and validation. J Chron Dis. 1987;40(5):373–83. 3558716 10.1016/0021-9681(87)90171-8

[33] Loya AM , González‐Stuart A , Rivera JO . Prevalence of polypharmacy, polyherbacy, nutritional supplement use and potential product interactions among older adults living on the United States‐Mexico border: a descriptive, questionnaire‐based study. Drugs Aging. 2009;26(5):423–36. 10.2165/00002512-200926050-00006 19552494

[34] Izzo AA , Ernst E . Interactions between herbal medicines and prescribed drugs: an updated systematic review. Drugs. 2009;69(13):1777–98. 19719333 10.2165/11317010-000000000-00000

[35] Gray SL , Hart LA , Perera S , Semla TP , Schmader KE , Hanlon JT . Meta‐analysis of interventions to reduce adverse drug reactions in older adults. J Am Geriatr Soc. 2018;66:282–8. 29265170 10.1111/jgs.15195 PMC5809283

[36] Nothelle SK , Sharma R , Oakes A , Jackson M , Segal JB . Factors associated with potentially inappropriate medication use in community‐dwelling older adults in the United States: a systematic review. Int J Pharm Pract. 2019;27(5):408–23. 30964225 10.1111/ijpp.12541 PMC7938818

[37] Hori S , Mihaylov I , Vasconcelos JC , McCoubrie M . Pattern of complementary and alternative medicine use amongst outpatients in Tokyo, Japan. BMC Complement Altern Med. 2008;8:14. 18433476 10.1186/1472-6882-8-14 PMC2375857

[38] Fonarow GC , Abraham WT , Albert NM , Stough WG , Gheorghiade M , Greenberg BH , et al. Factors identified as precipitating hospital admissions for heart failure and clinical outcomes. Arch Intern Med. 2008;168(8):847–54. 18443260 10.1001/archinte.168.8.847

[39] Agbabiaka TB , Savovic J , Ernst E . Methods for causality assessment of adverse drug reactions: a systematic review. Drug Saf. 2008;31(1):21–37. 18095744 10.2165/00002018-200831010-00003

[40] Ezaki H , Ayaori M , Sato H , Maeno Y , Taniwaki M , Miyake T , et al. Effects of Mokuboito, a Japanese Kampo medicine, on symptoms in patients hospitalized for acute decompensated heart failure – a prospective randomized pilot study. J Cardiol. 2019;74:412–7. 31272834 10.1016/j.jjcc.2019.05.003

