# Supplementary material for: The use of Kampo medications that may cause heart failure in hospitalized acute heart failure patients in a Japanese hospital
Source: J Gen Fam Med. 2020 Dec 9;22(3):141–7. doi: 10.1002/jgf2.411 (PMC8090841; doi:10.1002/jgf2.411)
Supplement: Supplementary file 1 — App S1 [file JGF2-22-141-s001.pdf]

Table S1. Type of Kampo medications included in this research.

**Kampo medication**	**Ingredients**	**Indications**
Shakuyakukanzoto	Peony, Licorice	Pain, myalgia or arthralgia, gastric pain and abdominal pain accompanied by sudden muscle spasms.
Junchoto	Rehmannia root, Angelica root, Scutellaria root, Immature orange, Apricot kernel, Magnolia bark, Rhubarb, Peach kernel, Hemp fruit, Licorice	Constipation.
Yokukansan	Poria sclerotium, Cnidium rhizome, Atractylodes rhizome, Uncaria hook, Angelica root, Bupleurum root, Licorice	Following symptoms of those patients with delicate constitution and nervousness: Neurosis, insomnia, night cry in children, and peevishness in children.
Juzentaihoto	Ginseng, Astragalus root, Atractylodes rhizome, Poria sclerotium, Angelica root, Peony, Rehmannia root, Cnidium rhizome, Cinnamon, Licorice	Declined constitution after recovery from disease, fatigue and malaise, anorexia, perspiration during sleep, cold limbs, and anemia.
Daikenchuto	Ginger, Ginseng, Zanthoxylum fruit	Abdominal cold feeling and pain accompanied by abdominal flatulence.
Keishininjinto	Cinnamon, Ginseng, Atractylodes rhizome, Licorice, Processed ginger	Following symptoms of patients with a weak digestive system: Headache, palpitation, chronic gastroenteritis, and gastric atony.
Rikkunshito	Atractylodes rhizome, Ginseng, Pinellia tuber, Poria sclerotium, Jujube, Citrus unshiu peel, Licorice, Ginger	Following symptoms of patients with weak stomach, loss of appetite and full stomach pit, and those who are easily fatigued, anemic and likely to have cold limbs: Gastritis, gastric atony, gastroptosis, maldigestion, anorexia, gastric pain, vomiting.
Yokukansanchinpihange	Bupleurum root, Uncaria hook, Atractylodes rhizome, Poria sclerotium, Angelica root, Cnidium rhizome, Citrus unshiu peel, Pinellia tuber, Licorice	Following symptoms of patients with a delicate constitution and nervousness: Neurosis, insomnia, night cry in children, and peevishness in children.
Shoseiryuto	Ephedra, Peony, Ginger, Licorice, Cinnamon, Asiasarum root, Schisandra fruit, Pinellia tuber	Following symptoms in persons with moderate or slightly poor physical strength who suffer from coughing with thin watery phlegm and runny nose: Bronchitis, bronchial asthma, rhinitis, allergic rhinitis, edema, common cold, and hay fever.
Bakumondoto	Ophiopogon tuber, Brown rice, Pinellia tuber, Jujube, Licorice, Ginseng	Following symptoms: Coughing with a hard, obstructive sputum, bronchitis, and bronchial asthma.
Mokuboito	Gypsum, Sinomenium stem, Cinnamon, Ginseng	Diseases originating from the heart or kidneys, as well as edema and cardiac asthma, of patients with a bad

		complexion, dyspnea with coughing, and tension and heaviness under the heart.
Byakkokaninjinto	Anemarrhena rhizome, Gypsum, Licorice, Ginseng, Brown rice	Thirst and hot flashes.
Ryokeijutukanto	Poria sclerotium, Cinnamon, Atractylodes rhizome, Licorice	Following symptoms in persons with moderate or poor physical strength who have dizziness and light-headedness, and sometimes experience hot flashes and/or palpitation: Syncope, dizziness, headache, ear noise, palpitations, shortness of breath, neurosis, and mental irritability.
Gosyuyuto	Jujube, Evodia fruit, Ginseng, Ginger	Following symptoms of patients with a moderately or less strong constitution who easily have cold hands and feet: Habitual migraine, habitual headache, vomiting, and cardiac beriberi.
Goshajinkigan	Rehmannia root, Achyranthes root, Cornus fruit, Dioscorea rhizome, Lantago seed, Alisma rhizome, Poria sclerotium, Moutan, Cinnamon, Aconite root	Following symptoms of patients with decreased urine volume or polyuria sometimes having dry mouth who are easily fatigued and easily feel cold in the extremities: Leg pain, low back pain, numbness, blurred vision in old patients, pruritus, dysuria, frequent urination and edema
Hochuekkito	Astragalus root, Astractylodes rhizome, Ginseng, Angelica root, Bupleurum root, Jujube, Citrus unshiu peel, Licorice, Cimicifuga rhizome, Ginger	Following symptoms in persons with poor physical strength who are out of energy with decline of gastrointestinal function and tend to get tired easily: Weak constitution, fatigue/malaise, weakness after illness or operation, loss of appetite, night sweat, and common cold.
Maoto	Ephedra, Apricot kernel, Cinnamon, Licorice	Following symptoms of patients with rigors, fever, headache, low-back pain, and skin without a sticky feeling: Common cold, influenza (in the initial phase), rheumatoid arthritis, asthma, nasal obstruction in suckling infants, and suckling difficulties.
Ninjinyoeito	Rehmannia root, Angelica root, Atractylodes rhizome, Poria sclerotium, Ginseng, Cinnamon, Polygala root, Peony, Citrus reticulate peel, Astragalus root, Licorice, Schisandra fruit	Following symptoms: Declined constitution after recovery from disease, fatigue and malaise, anorexia, perspiration during sleep, cold limbs, and anemia.

Table S2. Naranjo criteria.

	Yes	No	Do not know	Score
Are there previous conclusive reports on this reaction?	+1	0	0	
Did the adverse event appear after the suspected drug was administered?	+2	-1	0	
Did the adverse reaction improve when the drug was discontinued or a specific antagonist was administered?	0	0	0	
Did the adverse reaction reappear when the drug was re-administered?	+2	-1	0	
Are there alternative causes (other than the drug) that could on their own have caused the reaction?	-1	+2	0	
Did the reaction reappear when a placebo was given?	-1	+1	0	
Was the drug detected in the blood (or other fluids) in concentrations known to be toxic?	+1	0	0	
Was the reaction more severe when the dose was increased, or less severe when the dose was decreased?	+1	0	0	
Did the patient have a similar reaction to the same or similar drugs in any previous exposure?	+1	0	0	
Was the adverse event confirmed by any objective evidence?	+1	0	0	
			Total score[a]	

[a]The adverse drug reaction was assigned to a probability category from the total score as follows: definite more or 9, probable 5 to 8, possible 1 to 4, doubtful less than 1 (*Clin Pharmacol Ther* 1981;30:239-45).

Table S3. Results of the assessment of the causal link between the use of Kampo medications and acute heart failure in the 30 patients who used Kampo medications that may cause heart failure.

Case	Relevant composition of Kampo medications[a]	Naranjo score[b,c]	Causality[c]
#1	Licorice 13.5 g	3	Possible
#2	Licorice 0.7 g	5	Probable
#3	Licorice 1.0 g, ginseng 2.0 g	3	Possible
#4	Licorice 3.0 g, ginseng 4.5 g	3	Possible
#5	Licorice 0.5 g, ginseng 2.5 g	4	Possible
#6	Licorice 0.7 g, ginseng 2.7 g	3	Possible
#7	Licorice 2.0 g	3	Possible
#8	Licorice 1.5 g	3	Possible
#9	Licorice 2.0 g	3	Possible
#10	Licorice 1.0 g, ginseng 2.0 g	5	Probable
#11	Licorice[d]	2	Possible
#12	Licorice 1.0 g	3	Possible
#13	Licorice, ephedra	3	Possible
#14	Licorice 1.0 g, ginseng 4.0 g	3	Possible
#15	Licorice 1.3 g, ginseng 1.3 g	3	Possible
#16	Licorice 2.0 g, ginseng 4.5 g	3	Possible
#17	Licorice[d]	3	Possible
#18	Ginseng[d]	3	Possible
#19	Licorice 1.0 g	4	Possible
#20	Licorice 3.0 g, ephedra 3.0 g	3	Possible
#21	Licorice 0.3 g, ginseng 1.3 g	3	Possible
#22	Aconite 0.7 g	3	Possible
#23	Licorice 2.0 g, ginseng 0.7 g	3	Possible
#24	Licorice 1.5 g, ginseng 4.0 g	3	Possible
#25	Licorice 1.5 g, ephedra 5.0 g	4	Possible
#26	Licorice 0.5 g	3	Possible
#27	Licorice 0.5 g	6	Probable
#28	Licorice 4.0 g	4	Possible
#29	Licorice 2.0 g	3	Possible
#30	Licorice 2.0 g	6	Probable

[a]Dose per day.

[b]Based on the Naranjo criteria (*Clin Pharmacol Ther* 1981;30:239-45).

[c]Causality between Kampo medications and heart failure was assessed based on the Naranjo criteria. Adverse drug reactions were classified into four probability categories based on the total score: “definite” (more than 8 points), “probable” (5 to 8 points), “possible” (2 to 4 points), and “doubtful” (less than 2 points).

[d]Information on the dose of Kampo medications was not documented.

Table S4. Baseline characteristics of the 437 hospitalised patients with acute heart failure.

Characteristics	Total (n = 437)	Use of Kampo medications that may cause or exacerbate heart failure	
		No (n = 407)	Yes (n = 30)
Patient age			
Mean, SD	81.1 (12.1)	80.7 (12.3)	86.0 (7.8)
Median, IQR	84 (75 to 89)	84 (75 to 89)	87 (81 to 90)
Female sex	199 (45.5)	179 (44.0)	20 (66.7)
Japanese nationality	435 (99.5)	405 (99.5)	30 (100.0)
Nursing home resident	59 (13.5)	54 (13.3)	5 (16.7)
Charlson Comorbidity Index score			
Mean, SD	2.4 (1.7)	2.4 (1.7)	2.0 (1.6)
Median, IQR	2 (1 to 3)	2 (1 to 4)	2 (1 to 3)
Past medical history			
Stroke	96 (22.0)	89 (21.9)	7 (23.3)
Dementia	62 (14.2)	54 (13.3)	8 (26.7)
Diabetes mellitus	123 (28.2)	117 (28.8)	6 (20.0)
Ischaemic heart disease	66 (15.1)	64 (15.7)	2 (6.7)
COPD or asthma	52 (11.9)	50 (12.3)	2 (6.7)
Heart failure	239 (54.7)	228 (56.0)	11 (36.7)
Hypertension	355 (81.2)	329 (80.8)	26 (86.7)
Chronic kidney disease	208 (47.6)	194 (47.7)	14 (46.7)
Number of regular medications			
Mean, SD	6.2 (3.4)	6.1 (3.4)	7.4 (3.5)
Median, IQR	6 (4 to 8)	6 (4 to 8)	7 (5 to 10)
Medication use			
NSAIDs	23 (5.3)	22 (5.4)	1 (3.3)
Beta-blockers	141 (32.3)	137 (33.7)	4 (13.3)
ACE inhibitor or ARB	200 (45.8)	185 (45.5)	15 (50.0)
Loop diuretics	226 (51.7)	210 (51.6)	16 (53.3)
Spironolactone	64 (14.7)	60 (14.7)	4 (13.3)
Digoxin	12 (2.8)	11 (2.7)	1 (3.3)
Vital signs at admission			
Temperature	36.7 (7.0)	36.6 (0.7)	36.9 (0.8)

Systolic blood pressure, mmHg	146 (33)	146 (33)	147 (32)
Diastolic blood pressure, mmHg	86 (24)	86 (24)	83 (20)
Heart rate, beats per minute	96 (28)	96 (28)	96 (27)
Laboratory findings at admission			
Haemoglobin, g/dl	11.8 (2.1)	11.8 (2.2)	11.7 (1.8)
Blood urea nitrogen, mg/dl	29.2 (16.8)	29.2 (16.9)	30.0 (14.4)
Creatinine, mg/dl	1.3 (0.7)	1.3 (0.7)	1.2 (0.8)
Sodium, mmol/l	140 (8)	140 (8)	141 (5)
Potassium, mmol/l	4.3 (0.7)	4.3 (0.7)	4.1 (0.6)
Brain natriuretic peptide[b], ng/ml	1233 (1100)	1246 (1119)	1054 (813)
Triggers of heart failure			
Diet	100 (22.9)	93 (22.9)	7 (23.3)
Arrythmia	75 (17.2)	69 (17.0)	6 (20.0)
Uncontrolled hypertension	46 (10.5)	45 (11.1)	1 (3.3)
Infection	36 (8.2)	32 (7.9)	4 (13.3)
Cardiac ischaemia	28 (6.4)	28 (6.9)	0 (0.0)
Drug induced	19 (4.4)	18 (4.4)	1 (3.3)
Drug adherence	16 (3.7)	15 (3.7)	1 (3.3)
Anaemia	10 (2.3)	8 (2.0)	2 (6.7)
Exacerbation of CKD	6 (1.4)	6 (1.5)	0 (0.0)
Unknown	120 (27.5)	112 (27.5)	8 (26.7)
Heart vulvar disease			
None	138 (31.6)	133 (32.7)	5 (16.7)
Aortic valve stenosis	44 (10.1)	41 (10.1)	3 (10.0)
Aortic valve regurgitation	69 (15.8)	64 (15.7)	5 (16.7)
Mitral valve stenosis	2 (0.5)	2 (0.5)	0 (0.0)
Mitral valve regurgitation	190 (43.5)	174 (42.8)	16 (53.3)
Left ventricular ejection fraction[c], %	45 (17)	45 (17)	46 (19)
Duration of the index hospital stay, days			
Mean, SD	20.5 (16.6)	20.8 (17.0)	16.0 (10.1)
Median, IQR	16 (10 to 25)	16 (10 to 25)	17 (8 to 22)
In-hospital death	44 (10.1)	40 (9.8)	4 (13.3)

[a]Values are expressed as the number with the percentage of the total number, unless otherwise stated.

[b]Excludes 10 patients who were not tested for brain natriuretic peptide.

[c]Excludes 5 patients who were not evaluated for left ventricular ejection fraction.

ACE: angiotensin-converting enzyme; ARB: angiotensin receptor blocker; CKD: chronic kidney disease; IQR: interquartile range; NSAIDs: non-steroidal anti-inflammatory drugs; SD: standard deviation.
